# Editorial: Advances in Cell and Gene Therapy in Treating Neural Diseases

**DOI:** 10.3389/fncel.2021.794010

**Published:** 2021-11-15

**Authors:** Raymond Ching-Bong Wong, Kouichi Hasegawa, Gary S. L. Peh, Guei-Sheung Liu

**Affiliations:** ^1^Centre for Eye Research Australia, Royal Victorian Eye and Ear Hospital, Melbourne, VIC, Australia; ^2^Ophthalmology, Department of Surgery, University of Melbourne, Melbourne, VIC, Australia; ^3^Shenzhen Eye Hospital, Shenzhen University School of Medicine, Shenzhen, China; ^4^Institute for Integrated Cell-Material Sciences, Kyoto University, Kyoto, Japan; ^5^StemRIM Institute for Regeneration-Inducing Medicine, Osaka University, Osaka, Japan; ^6^Tissue Engineering and Cell Therapy, Singapore Eye Research Institute, Singapore, Singapore; ^7^The Ophthalmology & Visual Sciences Academic Clinical Programme (EYE-ACP), Duke-NUS Graduate Medical School, Singapore, Singapore; ^8^Menzies Institute for Medical Research, University of Tasmania, Hobart, TAS, Australia

**Keywords:** cell therapy, gene therapy, neural diseases, regenerative medicine, stem cells, exosomes

Neurological disorders affect both the central and peripheral nervous systems throughout the body, resulting in detrimental impacts on the patient's health and significant socio-economic burdens on our healthcare system. Often there is no effective means to cure neurodegenerative diseases. Recent developments in cell and gene therapy have induced a paradigm shift in the field of neuroscience, providing an innovative solution to develop treatments for neurodegenerative diseases. This Research Topic “*Advances in Cell and Gene Therapy in Treating Neural Diseases*” aims to provide a timely collection of original research articles, brief research reports, methods, perspectives and reviews in this exciting area. Capitalizing on the development of cell therapy, regenerative medicine, and gene therapy, their advances will help steer future research toward more efficient technologies and better treatment to address some of the most debilitating neurodegenerative diseases.

Cell-based therapy represents a promising strategy to treat neurodegenerative diseases. Grafted cells have the potential of modulating degenerative microenvironments in the nervous system; this in turn can help to halt tissue damage and/or promote regeneration. A research article by Xie et al. reported that grafted olfactory ensheathing cells (OECs), a unique type of glial cells, can delay degeneration in a retinal degenerative disease by the alleviation of activated resident microglia and reduction of inflammatory microenvironment. Zhai et al. further demonstrated that combined transplantation of neural stem cells (NSCs) and OECs, preserved the visual function and retinal structure in a rat model of retinal degeneration. As a result of this combined transplantation there was an increase in endogenous stem cell activation, better maintenance of NSC stemness, and enhancement in the migration of transplanted cells. Similarly using another type of stem cell—olfactory mucosa mesenchymal stem cells, Liu et al. demonstrated a neuroprotective effect by attenuating mitochondrial dysfunction and enhancing anti-oxidation in a rat model of cerebral ischemia/reperfusion (I/R). In addition, a review article by Barros et al. discusses the current treatments and strategies used to reduce polyQ symptoms and the major pre-clinical and clinical achievements obtained with mesenchymal stem cells transplantation, as well as obstacles that need to be overcome in order to translate this therapy in the clinic.

Apart from cell-based therapy, extracellular vesicles such as exosomes have also been widely explored for disease treatment. They show promising results in improving the recovery of structural and neurological functions in a disease setting. Exosomes play a pivotal role in mediating intercellular communication by delivering a variety of functional biomolecules to recipient cells. An article by Wooff et al. reveals that retinal small-medium extracellular vesicles (s-mEV) and their miRNA cargo play an essential role in maintaining retinal homeostasis through immune modulation. The authors demonstrate an inverse correlation between s-mEV concentration and photoreceptor survivability, as they observed a decrease in s-mEV numbers following photoreceptor degeneration. This knowledge provides a deeper insight to retinal degenerative diseases, in turn enabling better design of targeted therapy for retinal degeneration. Furthermore, an article published by Zeng Q. et al. investigates the effects of exosome derived from bone marrow-derived mesenchymal stem cells (BMSC-Exos) on I/R injury and determines if the mechanism is associated with the regulation of pyroptosis and autophagic flux. The findings indicate that BMSC-Exos can protect neural cells against I/R injury through the attenuation of NLRP3 inflammasome-mediated pyroptosis by promoting AMPK-dependent autophagic flux, furthering our understanding the pathological mechanism of cerebral I/R injury. Moreover, a meta-analysis by Huang et al. systematically describes the treatment of ischemic stroke with unmodified cell-derived exosomes. The result provides robust evidence that cell-derived exosomes can promote neurological recovery for individuals who suffered from a stroke.

Advances in induced pluripotent stem cell (iPSC) technology provides a feasible approach to generate patient-specific cells in the lab as an unlimited cellular source for tissue engineering and regenerative medicine. Numerous disease models have been developed using patient-specific stem cells to better understand disease pathogenesis, which also provided a personalized *in vitro* platform for drug discovery. Chen et al. successfully generated an iPSC line of sporadic juvenile amyotrophic lateral sclerosis (ALS) carrying a *de novo* pathogenic *FUS* mutation. The authors show that the cell line can be differentiated into motor neurons with pathological features of ALS. Another article by Guo et al. reports the use of iPSC to model an inherited retinal degenerative disease—retinitis pigmentosa with a novel *USH2A* mutation. By forming retinal organoids, the authors show that *USH2A* mutation causes disorganization in the neural retina and abnormalities in the retinal pigmented epithelium. This work demonstrates feasibility of the technology to recapitulate the pathogenesis of *USH2A* mutation using patient-derived retinal cells. Likewise, Chung et al. describe a new method of achieving retinal glial differentiation in human embryonic stem cells promoted by Notch signaling. The methodology can help advance the generation of stem cell disease models to study the pathogenesis of retinal diseases associated with glial dysfunction. Moreover, a review article by Hayashi et al. highlights the use of patient-derived iPSCs carrying chromosomal abnormality for future studies on elucidating pathogenesis and therapeutics development for abnormal chromosomal diseases including neurodevelopmental diseases.

Recent preclinical studies and on-going clinical trials have demonstrated the promising potential of using gene editing-based therapy to correct the underlying genetic defects that cause inherited diseases. Research into the safety, specificity, and efficacy of gene editing therapy would be key to facilitate its translation from the bench to the clinics. Li et al. compare various CRISPR/Cas-based gene editing systems for *in vivo* gene editing of neurosensory retinal cells. The results indicate that the adeno-associated virus-mediated delivery of the CRISPR/SpCas9 construct achieves the most efficient gene modification. Another article by French et al. review the research progress of developing commercial gene- or cell- therapy for patients with Usher syndrome. The article also highlights the importance of improving the safety and efficacy of gene therapy approaches in order to provide treatment options for Usher syndrome patients.

Understanding the cellular and molecular changes of neurological diseases is a critical step for developing a better therapeutic approach. In the article by Yu et al., the team explores the pathogenesis of deafness and its association with the *Fgf13* mutation. The study reveals the novel role of *Fgf13* in auditory function, in which it regulates the survival of spiral ganglion neurons in the inner ear making it a potential drug target for treating deafness. Another article by Zeng S. et al. reviews the latest research on interphotoreceptor retinoid-binding protein (IRBP), a lipophilic glycoprotein specifically secreted by photoreceptors. This review discusses the potential of manipulating the expression of IRBP to rescue or prevent photoreceptor degeneration in retinal diseases. Furthermore, a review article by Yang et al. explores research on the role of endothelial progenitor cells (EPCs) in depression, in particular focusing on the potential of using EPCs as a new target for evaluating the severity of depression. Similarly for epilepsy, in an original article, Zhang et al. explored the neuroprotective effect of Xenon as a potential intervention for seizures and epilepsy.

The presented article collection in this Research Topic covered a broad range of topics to offer profound insight in the development of cell and gene therapy for neural diseases, which should be of high interest to many specialists in the neuroscience field.

## Author Contributions

All authors contributed to conceptual design, writing, and approval of the manuscript.

## Conflict of Interest

The authors declare that the research was conducted in the absence of any commercial or financial relationships that could be construed as a potential conflict of interest.

## Publisher's Note

All claims expressed in this article are solely those of the authors and do not necessarily represent those of their affiliated organizations, or those of the publisher, the editors and the reviewers. Any product that may be evaluated in this article, or claim that may be made by its manufacturer, is not guaranteed or endorsed by the publisher.

